# Potential risk factors and triggers for back pain in children and young adults. A scoping review, part II: unclear or mixed types of back pain

**DOI:** 10.1186/s12998-019-0281-8

**Published:** 2019-11-19

**Authors:** Amber M Beynon, Jeffrey J Hebert, Charlotte Lebouef-Yde, Bruce F Walker

**Affiliations:** 1 College of Science, Health, Engineering and Education, Murdoch University, 90 South Street, Murdoch, 6150 Western Australia Australia; 2 Faculty of Kinesiology, University of New Brunswick, 3 Bailey Drive, Fredericton, New Brunswick E3B 5A3 Canada; 3 Institute for Regional Health Research, University of Southern Denmark, Odense, Denmark

**Keywords:** “Risk factors”, “Back pain”, Children, Adolescent, Young adult, Scoping review

## Abstract

**Background:**

Back pain is a global problem in terms of disability and financially, with a large burden both to the individual and to society. Back pain was previously believed to be uncommon in children. However, there is a growing body of evidence that this is not the case.

**Objective:**

Part I of this scoping review studied risk factors of incident and episodic back pain. In this part II we aimed to identify all risk factors and triggers with unclear or mixed type back pain in young people and to identify any gaps in the literature.

**Methods:**

A scoping review design was selected to summarise the evidence, as there are many studies on “risk factors” for back pain. The scoping review followed the PRISMSA-ScR guidelines. We considered all studies that tested potential risk factors and triggers for thoracic and/or lumbar spine pain, in children, adolescents, and young adults (≤ 24 years). PubMed and Cochrane databases were searched from inception to September 2018, to identify relevant English language articles. The results regarding potential risk factors were separated into temporal precursors and bidirectional risk factors and the studies were classified by study design.

**Results:**

Our comprehensive search strategy identified 7356 articles, of which 83 articles were considered eligible for this review (part II). There were 53 cross-sectional studies and 30 cohort studies. Potential risk factors for back pain were: female sex, older age, later pubertal status, positive family history of back pain, increased growth, and a history of back pain, most of which are temporal precursor variables. There was limited research for the illness factors, spinal posture, and muscle endurance in the development of back pain.

**Conclusion:**

Many of the included studies approached risk factors in similar ways and found factors that were associated with back pain but were not obvious risk factors as causality was uncertain. Future research should be more rigorous and innovative in the way that risk factors are considered. This could be through statistical approaches including cumulative exposures, or longitudinal approaches including multi-trajectory methods. Additionally, data on proposed risk factors should be collected before the onset of back pain.

## Background

Back pain is a global problem in terms of disability and financial costs, with a large burden both to the individual and to society [1]. Back pain was once believed to be uncommon in young people. However there is evidence that this is not the case [2, 3]. Back pain can start during childhood or adolescence [2, 3]. Therefore, it is important not to ignore younger populations. Numerous studies have attempted to investigate a myriad of potential risk factors of back pain in children and young adults. Identifying early life factors that predispose young people to back pain in later life may help identify at-risk populations and inform future prevention strategies. Prevention of back pain in adolescence could help the prevention of back pain into adulthood [4].

Some potential risk factors definitely occur before the inception of the disease; we define these variables as temporal precursors. Temporal precursors are variables known to have a definite preceding temporal relationship with a disease (e.g., sex, age, pubertal status, family history, family socioeconomic factors, and height). Conversely, other factors studied may not have occurred prior to the onset of the disease, and they can have a bidirectional relationship with the disease of interest. If such potential risk factor is measured concurrently with back pain, then we cannot know if the potential risk factor preceded the back pain or not. Examples include body mass index (BMI), muscle endurance and flexibility, posture, physical activity behaviour, work, screen time, inadequate sleep, smoking, illnesses, and psychosocial factors.

Due to the vast number of studies on “risk factors” for back pain a two part scoping review of the literature was chosen as the best way to summarise the evidence. Part I of this scoping review *(Potential risk factors and triggers for back pain in children and young adults. A scoping review, part I: incident and episodic back pain)* studied risk factors of incident and episodic back pain. In Part II we aimed to identify all risk factors and triggers for back pain (unclear or mixed types of back pain) in young people and to identify any gaps in the literature. Moreover, in this second part, all eligible studies (unclear or mixed types of back pain) that tested potential risk factors of back pain and triggers of its further episodes were included.

## Methods

**Fig. 1:**
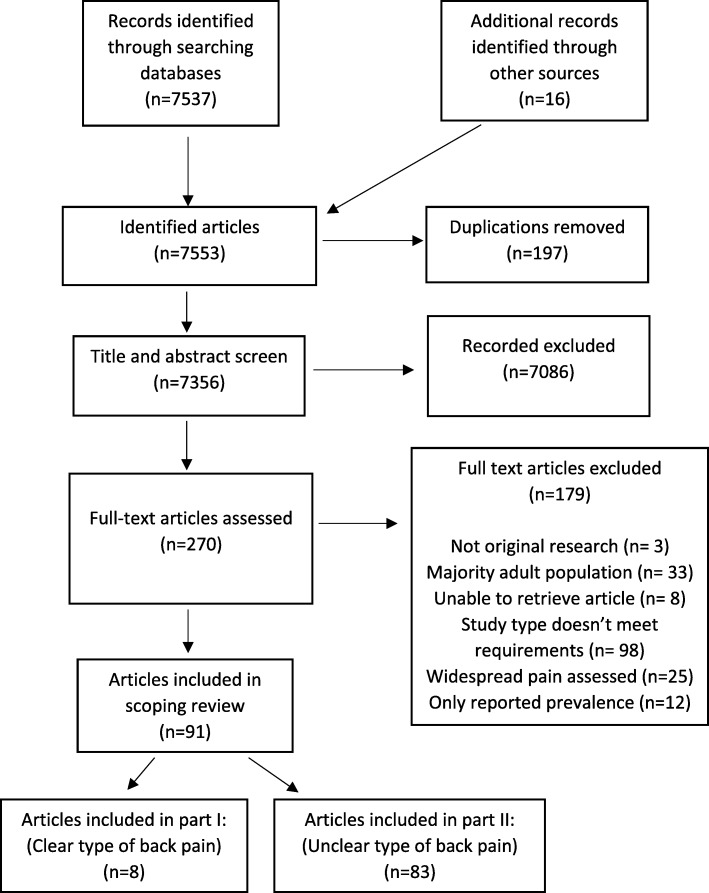
Final study selection flow diagram

The full methods are reported elsewhere *(Potential risk factors and triggers for back pain in children and young adults. A scoping review, part I: incident and episodic back pain).* However, a summary of the methods is provided below. We undertook a scoping review in accordance with reporting guidelines (PRISMA-ScR) [5]. A review protocol was not included in a registry as PROSPERO does not currently accept registrations for scoping reviews. The broad question of interest was *what are the potential risk factors and potential triggers for back pain in childhood and young adulthood?* ‘Back pain’ was defined as pain within the thoracic and/or lumbar areas. A search was conducted using the PubMed and Cochrane databases from inception to September 2018. The full search strategy is listed in Additional file 1. Results of the search were reported as per the PRISMA flow diagram (Fig. 1).

### Eligibility criteria

We included studies that reported on potential risk factors or triggers for pain in the thoracic and/or lumbar spine (a risk factor is the cause of ‘disease’ of back pain defined as the first time they have back pain compared to a trigger, which could lead to an episode of back pain when the disorder of back pain is already established). The majority of the participants were to be less than 25 years old at baseline. The age classification is based on the MeSH definition of a young adult (19–24 years). Additionally, the contemporary definitions of adolescence includes young adulthood (10–24 years) [6]. Original peer-reviewed studies in the English language from any country of origin were included and study designs comprised retrospective, cross-sectional, and prospective observational studies. Cross-sectional studies were only included if the potential risk factors met Bradford Hill’s tenet of temporality for the study of risk factors or triggers (i.e., if the exposure was classified as a temporal precursor e.g. age) [7].

### Study selection, data charting and synthesis of results

Titles, abstracts, and full-text articles were screened by one researcher (AB) twice (March 2018 and then September 2018) against the inclusion criteria. The second search identified four additional articles due to the passage of time. Another researcher (BW) verified the study selection for accuracy (titles, abstracts, and full-text screen) and full consensus was met through discussion.

Calibration of the data charting forms was conducted by two researchers (AB and CLY). One researcher (AB) piloted the form on three studies. This process was verified by another researcher (CLY). This was an iterative process in which there were many changes during each round. Any disagreements were resolved by a third researcher (BW).

Charting of data (data extraction in scoping reviews [8]) was completed by one researcher (AB) using the evidence tables. This information was checked for errors several times with an audit of all data entered with at least a week between each audit. Potential risk factors or triggers were separated into temporal precursors or potentially bidirectional risk factors. Results for the cross-sectional and prospective studies are reported together for potential risk factors that are inherently present before the back pain (temporal precursors). If a study had multiple estimates for the same risk factor the most adjusted estimate of association was extracted. Clarity of definition of back pain was assessed in each study with a summative score provided. Individual points were given if there was a clear description of the area of back pain, a clear reporting of the recall period, a clear definition of the type of back pain, and if there was an attempt to collect valid data (maximum four points).

## Results

### Study selection

Our database searches identified 7537 articles and a subsequent search of the relevant references lists resulted in an additional 16 articles. In all, 91 articles were considered eligible for this review. Eight studies appeared to have studied risk factors of incident back pain and back pain episodes (reported in part I). Within part II, 83 studies were included, as these failed to clearly identify whether they studied inception events or ongoing/episodes of back pain (Fig. 1).

### Study characteristics and synthesis of results

Of the 83 articles included in this review, 30 (36%) were prospective cohort studies [4, 9–37]. The majority of cohort studies did not have a clear description of back pain or captured a mixture of back pain types. Thus, many studies appear to have dealt with either back pain episodes or the incidence of back pain. They only considered a limited time frame and did not report details of the previous pain-free period. Therefore, these studies could reflect a mixture of first time, recurrent, and ongoing back pain episodes.

The included studies included temporal precursor variables such as sex, age, pubertal status, family history, socioeconomic status, and height. Potential bidirectional variables included BMI, muscle endurance and flexibility, posture, physical activity and work, screen time, inadequate sleep, carrying bags, smoking, illnesses, and psychosocial factors. Charts of the summary of findings are reported in Additional file 2.

There were 53 cross-sectional studies included in this review [38–90]. These studies reported factors that could potentially be associated with back pain such as sex, age, pubertal status, family history, and socioeconomic status. Charts of the summary of findings are seen in Additional file 3.

### Temporal precursor variables

#### Sex

In the 53 studies reporting on sex and back pain, 32 studies found a positive association with female sex and back pain, three studies found a higher prevalence of back pain in males, and 18 studies found no association with sex (Table 1). There was generally a positive association between female sex and back pain.

**Table 1:** Summary of temporal precursor variables: cross-sectional and prospective studies

Variable	Number of studies	Number of studies: Increased risk	Number of studies: Decreased risk	Number of studies not significant	Strength of association (95% CI)	
Female sex	53	32	3	18	*Positive association:* OR 1.9 (1.4, 2.0) (c) [10] OR 1.9 (1.4, 2.4) (c) [16] OR 2.4 (1.9, 3.1) (LBP), OR 2.2 (1.6, 2.9)(MBP) (c) [17] OR 1.6 (1.2, 2.0) [26] OR 1.7 (1.4, 2.1) (c) [28] OR 1.6 (1.4, 2.0) (c) [29] OR 7.7 (4.7, 12.6)) [34] OR 1.7 (1.5, 2.0) [39] OR 1.3 (1.4, 3.3) [42] OR 1.5 (1.2, 1.8) [43] OR 2.2 (1.4, 3.3) [44] OR 1.5 (1.1, 1.9) [45] OR 1.5 (1.1, 1.9) [47] OR 2.4 (1.7, 3.3) [51] OR 1.5 (1.0, 2.1) [53] OR 2.1 (1.6, 2.9) [54] OR 1.4 (1.0, 2.1) (c) [58]	OR 1.1 (1.1, 1.2) [59] OR 1.9 (1.7, 2.2) [64] OR 2.1 (1.6, 2.7) [66] PR 1.1 (1.1, 1.2) [69] PR 1.2 (1.1, 1.3) [70] OR 1.6 (1.3, 2.1) [74] OR 1.8 (1.2, 2.7) (c) [76] OR 4.6 (1.8, 11.7) [78] OR 2.2 (1.6, 2.9) [77] OR 2.4 (1.9, 3.2) [79] OR 1.6 (1.3, 2.0) (c) [81] OR 1.8 (1.3, 2.4) (c) [83] OR 2.7 (1.2, 6.1) [84] Females: 28%, Males 19% [85] OR 1.9 (1.3, 3.0) [89] *Negative association:* Males: HR 3.2 (2.7, 3.7) [27] OR 0.6 (0.4, 0.8) [50] OR 0.3 (0.2, 0.5) (c) [68]
Older Age	34	19	2	13	*Positive association:* OR 2.9 (2.6, 3.3) (c) [32] OR 1.5 (1.1, 2.3) [35] OR (17 index), 21 yr 2.2 (1.2, 4.2), 23 yr 3.2 (1.7, 6.2), 24 yr 2.8 (1.5, 5.3) [42] OR (10–11 index), 12–14 yr: 1.1 (1.1, 1.3) [47] OR 1.1 (1.1, 1.2) [51] (15 index) 16/17 yr OR 1.7 (1.2, 2.3), 18/19 yr: OR 1.8 (1.2, 2.8) [53] 14 to 15 yr: 6.4% increase [54] OR 1.2 [58] *r* 0.2 [61] (17/18 index), 21+ yr: OR 1.6 (1.2, 2.1) [65] (10–12 index), 13–16 yr: OR 1.5 (1.2, 2.0) [66] (per year): OR 1.2 (1.1, 1.4) [72] OR 1.2 (1.1, 1.3) [74] Older 25.1%, younger adolescents 12.4% [75] (12 index), 14 yrs.: OR 1.3 (1.1, 1.7) [80] Younger age: OR 1.5 (males), OR 1.4 (females) [81] 11 yr 18%, 14 yr 34% (girls) 11 yr 14%, 14 yr 25% (boys) [85] OR 1.3 (1.1, 1.7) [88] OR 1.3 (1.2–1.4) [89] *Negative association:* Younger age: OR 0.2 (0.1, 0.6) [46] OR 0.5 (0.4, 0.6) [67]	
Positive family history	19	15	0	4	OR 3.6 (1.3, 10.2) [11] OR 2.1 (1.4, 3.1) [35] OR 2.0 (1.1, 4.0) [36] OR 2.6 (1.4, 5.9) [38] OR 2.1 [40] OR 3.8 (2.9, 5.9) [41] OR 1.8 (1.4, 2.4) [43] OR: 1.5 (1.1, 1.9) (c) [48]	OR 1.7 [58] OR 1.8 (1.5, 2.0) [64] PR 1.2 (1.2, 1.3) [69] PR 1.2 (1.1, 1.3) [70] OR 2.0 (1.2, 3.3) [72] OR 2.3 (1.2, 4.7) [89] OR 2.6 (1.9, 3.6) [90]
Socioeconomic factors	15	7	0	8	Higher Socioeconomic index: OR 0.8 (0.7, 1.0) [34] Higher social class: OR: 0.9 (0.8, 0.9) [55] Parental low level of education: OR 1.8 (1.1, 2.0) [62] Ethnicity: (Index white) Asian PR: 1.2 (1.1, 1.4), indigenous PR: 1.4 (1.3, 1.5) [70] Non-white: PR 1.4 (1.0, 1.9) [71] Location (index peripheral center) Urban centre: OR 3.1 [73] Residence: 52% (city), 43% (village) [83]	
Increased height or increased growth spurt	12	4	1	7	High growth spurt: OR 3.1 (1.5, 6.0) [4] linear growth: IRR 1.2 (1.2, 1.2) [18] Shorter than median height (158 cm): RR 2.1 (1.2, 3.8) [23] Height: OR 1.2 (1.0–1.5) [31] Taller: *t* test − 3.3 [58]	
Later pubertal status	6	4	1	1	*Positive association* IRR 1.5 (1.2, 2.0) (Tanner stage 2), IRR 2.1 (1.5, 3.0) (Tanner stage 3) IRR 3.3 (2.1, 5.0) (Tanner stage 4/5) [18] OR 1.6 (1.3, 2.0) (USA), OR 1.3 (1.1, 1.6) (Dutch) [22] OR 2.0 (girls), OR 1.9 (boys) [63] Stage 4: OR 2.0 (1.3, 3.5), stage 5: OR 2.1 (1.1, 1.4) [86] *Negative association:* HR 0.6 (0.5, 0.8) (males) [27]	
History of back pain	3	3	0	0	BP in adolescence for BP in adulthood: OR 4.3 (3.5, 5.4) [21] History of BP: OR 2.7 (1.1, 7.1) (ever), OR 9.1 (3.0, 27.2) (> 7 days) [33] History of BP: OR 7.7 (4.7–12.6) (girls) [34]	

*OR* odds ratio, *PR* prevalence ratio, *HR* hazard ratio, *RR* relative risk, *IRR* incidence rate ratio, *LBP* low back pain, *MBP* mid back pain, *BP* back pain, (c): parameter measure calculated from the provided results within study i.e. percentages converted to odds ratios

#### Age

In the 34 studies reporting on age and back pain (Table 1), there was generally a higher prevalence of back pain with advancing age in children towards adolescence and young adulthood.

#### Family history

In the 19 studies reporting on family history and back pain (Table 1), there was by and large a higher prevalence in those with a positive family history of back pain.

#### Socioeconomic status

In 15 studies there were inconsistent estimates of association for the relationship between socioeconomic factors and back pain. Seven studies reported positive associations between certain socioeconomic factors and back pain, whereas eight studies reported no association (Table 1).

#### Increased height or increased growth spurt

In the 12 studies on height or increased growth there were inconsistent estimates of association for the relationship between these and back pain (Table 1). Overall height does not appear to be a risk factor for back pain. However, the occurrence of ‘growth spurts’ has been found to be positively associated with back pain.

#### Pubertal status

As demonstrated in Table 1, in the six studies that reported on pubertal status and back pain, there was an association with back pain typically seen in those with an advanced pubertal status.

#### History of back pain

Three studies reported on history of back pain and risk of further back pain (Table 1). All studies found a positive association with odds ratios ≥2.7.

### Bidirectional variables

#### Physical activity and work

Ten studies considered physical activity and/or work as a potential risk factor of back pain. Six studies reported that with certain types of physical activity or work there was an increased prevalence of back pain, whereas four studies found no association (Table 2). It appears certain types of work such as white-collar work or manual work, and vigorous or high levels of physical activity may be associated with back pain.

**Table 2:** Summary of bidirectional variables

Variable	Number of studies	Number of studies: Increased risk	Number of studies: Decreased risk	Number of studies not significant	Strength of association (95% CI)
Physical activity/work	10	6	0	4	Playing sport OR 9.5 (1.9, 48.2) [11] White collar work OR 4.9 (1.7, 14.2) [13] Vigorous intensity physical activity: OR 1.2 (1.0–1.4) (diagnostic spinal pain) OR 1.3 (1.0–1.5) (traumatic) [15] High level sports activity RR 1.6 (1.1, 2.3) [24], Part-time work RR 1.5 (1.1, 2.1) [24] Provoked by manual work: OR 9.2 (2.9, 28.8) [33] Increased physical activity OR 1.9 (1.2, 2.8) [34]
Psychological factors	7	4	0	3	High level of peer problems: RR 2.3 (1.3, 4.2) [23] High level of psychological factors: RR 1.6 (1.1, 2.3) [24] Externalising behaviour: RR 1.5 (1.3, 1.7) (boys), RR 1.4 (1.3, 1.5) (girls), RR 3.6 (1.5, 8.5) (girls 18) [28] High levels of aggressive behaviour OR 1.4 (1.2, 1.6) [34] High level of somatic complaints OR 1.3 (1.1, 1.5) [34]
Higher BMI	8	3	0	5	OR 1.3 (1.0, 1.5) [11] RR 1.1 (1.0, 1.2) (girls), RR 1.1 (1.0, 1.3) (boys) [29] OR 2.9 (1.7, 5.1) (9 yr), 2.2 (1.4, 3.5) (10 yr), 1.6 (1.2, 2.1) (13 yr) [32]
Smoking	6	6	0	0	OR 2.2 (1.4, 3.5) [4] OR 2.4 (1.3, 6.0) [14] OR 3.1 (1.1, 9.2) (MB), 1.8 (1.2, 2.8) (BP) [17] OR 1.7 (1.4, 2.1) [19] HR 1.6 (1.4, 1.9) [27] OR 2.5 (1.4, 4.5) (females) [30]
Illness	4	3	0	1	Asthma OR 1.4 (1.1, 1.7) (female) [20] Headache OR 1.6 (1.1, 2.1) (female), OR 2.4 (1.2, 4.7) (male) [20] Abdominal pain RR 1.8 (1.1, 3.0) [24] Headache OR 2.4 (1.8, 3.1) [26]
Posture/sitting position	4	4	0	0	No LB support: OR 1.7 (1.2, 2.6), OR 2.9 (1.1, 3.5) (persistent LBP) [25] Provoked by sitting OR 3.8 (1.3, 11.3) [33] Non-neutral standing posture OR 2.2 (1.3, 3.6) [34] Uncomfortable school desk OR 6.0 (3.7, 9.7) [35]
Insufficient sleep	3	3	0	0	OR 2.9 (1.7, 5.2) (girls), OR 2.4 (1.3, 4.5)(boys) [10] OR 2.2 (1.7, 3.8) [35] OR 1.2 (1.1, 1.4) [36]
Flexibility	3	2	0	1	Decreased flexibility: hamstrings OR 1.1 (1.0, 1.1) [4] Decreased flexibility: quad muscles: OR 1.7 (1.1, 2.8) [25]
Screen time	3	1	0	2	Increased TV time OR 2.0 (1.4, 2.9) [35]
Backpack factors	3	1	0	2	Heavy school satchel OR 2.2 (1.0, 4.8) [36]
Muscle endurance	1	1	0	0	Poor back muscle endurance OR 1.9 (1.2, 3.0) [34]

*OR* odds ratio, *RR* relative risk, *HR* hazard ratio, (c) parameter measure calculated from the provided results within study i.e. percentages converted to odds ratios

#### Psychosocial factors

In the seven studies that tested psychosocial factors as risk factors of back pain, four studies found an increased risk of back pain, while three studies found no association (Table 2). Some psychosocial factors (depression, anxiety and ‘peer problems’) were associated with back pain while internalising, anxiety sensitivity, dysfunctional coping, and catastrophizing were not associated with future back pain.

#### Body mass index

In the eight studies that reported on BMI and back pain (Table 2), three studies reported an increased prevalence and five studies found no association (Table 2). There were inconsistent estimates of association, with insufficient evidence to conclude that there is a relationship between BMI and back pain.

#### Smoking

In the six studies that reported on smoking and back pain (Table 2), all found a positive association between the two. It does appear that smoking has some relationship with back pain.

#### Systemic factors /illnesses

Four studies tested systemic factors or illnesses as potential risk factors of back pain. Three studies found positive associations whereas one found none (Table 2). Associations with back pain were stronger with certain systemic diseases such as having asthma, headaches, abdominal pain, and colds/minor illnesses. These may be co-morbidities to back pain, meaning that one could be a precursor to the other or they could have a common cause.

#### Spinal posture and sitting posture

Four studies reported on certain aspects of posture and back pain (Table 2). All four studies indicated that from a preliminary viewpoint abnormal spinal posture and certain sitting positions were associated with back pain.

#### Sleep

As seen in Table 2, in the three studies that reported on sleep and back pain, there was a positive association between back pain and insufficient sleep.

#### Flexibility

Three studies tested muscle flexibility as a risk factor for back pain (Table 2). Two studies found a positive association with decreased flexibility of hamstrings or quadriceps, and back pain, while one study found no association.

#### Screen time

Three studies reported inconsistent estimates of associations between screen time and back pain. One study reported a higher prevalence of back pain with increased television time, whereas two reported none (Table 2).

#### Backpack factors

In three studies, there were inconsistent estimates of association between backpack factors and back pain. One study of these three reported a higher prevalence of back pain with a heavier school satchel (Table 2).

#### Muscle endurance

In the one study that tested muscle endurance as a risk factor of back pain, it was found that those with poor back muscle endurance had a positive association with back pain (Table 2).

## Discussion

### Overall summary of potential risk factors from all studies

Considering the existing literature, the factors found to be likely risk factors or triggers for back pain are female sex, older age, advanced pubertal status, high growth rate, positive family history of back pain, a history of back pain, smoking, and insufficient sleep. Most of these factors are temporal precursor. Further, they are mostly biological and non-modifiable, making them ineligible targets for preventative interventions. No association or weak associations were noted with increased screen time and work. There were mixed results for muscle flexibility, socioeconomic status, backpack-related factors, anthropometric measures including height and weight, and physical activity. There was limited research for systemic/illness factors, muscle endurance, spinal posture, and sitting position (Table 3).

**Table 3:** Summary of Potential Risk factors

Potential risk factor	Likely	Weak/no significance	Mixed results/ inconsistent	Limited research
Female sex	X			
Older age/ advanced pubertal status	X			
Positive family history of back pain	X			
Increased growth spurt	X			
History of back pain	X			
Smoking	X			
Insufficient sleep	X			
Increased screen time		X		
Work		X		
Psychosocial factors			X	
Muscle flexibility			X	
Socioeconomic status			X	
Backpack related factors			X	
Height and weight			X	
Physical activity			X	
Spinal posture				X
Sitting position				X
Systemic/illness factors				X
Muscle endurance				X

### Implications of results

Previous systematic reviews found the most likely risk factors for back pain in young people to be female sex [91–93], older age [91, 92, 94], advanced pubertal status [95], positive family history of back pain [96], and a previous history of back pain [93, 97]. We advanced this knowledge by further considering the temporal relationship between the risk factors and back pain and we concluded that the most likely risk factors or triggers for back pain are predominantly biological. For example, the genetic component of back pain is potentially large [98]. A systematic review found that estimates of heritability effects ranged from 21 to 67% [99]. However, environmental exposures also have an effect, so the question arises; how large is this effect? This question could be addressed through further twin control studies. Twin studies have an advantage of reducing confounding due to genetics and can be utilised to explore the potential causal pathway between environmental factors, co-morbidities and back pain [99].

Considering the strength of associations, some factors were statistically linked to back pain, but the next question arises, are they important on a clinical or individual level?

Another issue to consider is that individual associations may well be relatively weak, but it is possible that combination of factors or the addition of factors could increase the risk of back pain rather than individual factors. This idea has been proposed previously through a dynamic multifactorial and recursive model of aetiology [100]. This model emphasizes the importance of investigating intrinsic predisposing factors along with the extrinsic factors that interact together to make an individual vulnerable to injury [100]. Certain predictive risk factors could predispose individuals to back pain, and then in combination with other potentially causal risk factors, the individual could develop back pain. For example, girls (factor 1) with advanced pubertal status (factor 2) could be susceptible to back pain that is subsequently caused by vigorous physical activity (factor 3). Therefore, from a clinical perspective, it might be important to consider the person as a whole.

### Limitations of the current literature

The foremost limitations of the current literature are that the majority of studies are cross-sectional, or if longitudinal, most do not start data collection before the onset of back pain. To investigate temporality, one criterion to establish causal relationships, risk factors should be captured before the inception of the disease [101]. Therefore, the conclusions of this scoping review are limited to demonstrating association and not causation. Additionally, the definitions of ‘back pain’ vary from study to study (Additional files 4 and 5), this means it is not clear whether authors are considering back pain as a disease or an episode [101], or whether they are asking about back pain currently, for the past week, for the past month, or for the past year. Although the purpose of this review was to include risk factors or triggers for pain the thoracic and/or lumbar spine, some of the included studies included spinal pain in general [9, 15, 16, 18, 35, 39, 86–89]. While other studies included back pain without a clear definition of location [22, 26, 49, 50, 56, 61, 63, 69, 70, 74, 81], and therefore it is unclear what they were looking at. As the definitions on back pain are not always clear or inconsistent it is difficult to make clear definitive statements.

### Limitations of this review

A potential limitation of the validity of data collected in this scoping review is that only one researcher screened and conducted data charting. Nevertheless, articles were screened twice, by the same reviewer, and a second researcher verified the process, which is consistent with the PRISMA-ScR guidelines. Only two key databases were searched, and articles were limited to English language. Consequently, we may have missed some articles. Nevertheless, this type of literature is quite stereotyped, for which reason it is unlikely that any missing articles would be of significance.

There were some contradictory findings in our result tables. Contradictory findings often result from differences in study populations, definitions of the outcome or independent variables, and differences in data quality. However, due to the nature of scoping reviews, which lack the critical approach of systematic reviews, such contradictions cannot be interpreted. Due to the nature of conflicting data the summary of potential risk factors is indicative but not unequivocal.

### Recommendations for future research

Future studies should collect data from the inception of back pain by following the population from earlier life, if searching for causes of the ‘disease’ back pain. They should additionally collect data on proposed risk factors before the onset of back pain. If studies are attempting to identify triggers of future events, back pain episodes must be separated by non-episodes.

As highlighted within this scoping review (Additional files 4 and 5), future research should ensure that data are collected with a clear definition of back pain and ideally measured through a validated questionnaire. Additionally, future research should be more innovative in the way that risk factors are considered. This could be through statistical approaches including cumulative exposures, or longitudinal approaches such as multi-trajectory methods and through the use of twin studies.

## Conclusion

Many of the included studies approached identifying risk factors in similar ways and found factors that were associated with back pain but were not obvious risk factors as causality was uncertain. Obviously, the time has come to approach this problem in other ways. It is our opinion that future research should be more rigorous and innovative in the way that risk factors for back pain are considered.

## Supplementary information

**Additional file 1.** Search strategies used for the literature search. The full search strategy for PubMed and Cochrane databases.

**Additional file 2.** PROSPECTIVE STUDIES reporting factors that are longitudinally associated with back pain. Table summarising each included prospective study.

**Additional file 3.** CROSS-SECTIONAL STUDIES reporting factors that are associated with back pain. Table summarising included cross-sectional study.

**Additional file 4.** Clarity of definitions of Back pain: Prospective studies. Table summarising the clarity of the definitions of back pain in included prospective studies.

**Additional file 5.** Clarity of definitions of Back pain: Cross-sectional studies. Table summarising the clarity of the definitions of back pain in included cross-sectional studies.

## Data Availability

Not applicable.

## Abbreviations

BMI: Body mass index
BP: Back pain
CI: Confidence intervals
HR: Hazard ratio
IRR: Incidence rate ratio
LBP: Low back pain
MBP: Mid back pain
N: Number of participants
NA: Not applicable
OR: Odds ratio
PR: Prevalence ratio
RR: Relative risk
