# Supplementary material for: Potential risk factors and triggers for back pain in children and young adults. A scoping review, part II: unclear or mixed types of back pain
Source: Chiropr Man Therap. 2019 Nov 19;27:61. doi: 10.1186/s12998-019-0281-8 (PMC6862810; doi:10.1186/s12998-019-0281-8)
Supplement: Supplementary file 2 — Additional file 2. PROSPECTIVE STUDIES reporting factors that are longitudinally associated with back pain. Table summarising each included prospective study. [file 12998_2019_281_MOESM2_ESM.pdf]

Additional file 2: PROSPECTIVE STUDIES reporting factors that are longitudinally associated with back pain.

Back pain, sample characteristics and associations between back pain and temporal precursor variables. Prospective Studies

**Reference**	**Back pain**					**Characteristics of study sample**		**Significant positive (+) or negative (-) associations with back pain**								
**Ref, (year of pub), country, pop size**	**MBP**	**LBP**	**Mix**	**?**	**Clear definition of BP (x/4)** (Additional file 4)	**Age range (mean)**	**Sex**	**Female**	**Male**	**Age**	**Pubertal status**	**Family history BP**	**Socioeconomic factors**	**Height**	**History of back pain**	**Significant estimates (95% CI)**
[10] Auvinen, (2010), Finland, 1773		X			2/4	15-16	Both	+		(NT)	(NT)	(NT)	(NT)	(NT)	(NT)	Female: OR 1.7 (1.4-2.0) (age 16), OR 1.9 (1.6-2.3) (age 18) (calculated)
[11] Balague´, (2010), Switzerland, 95		X			2/4	13-14 (14.0)	Male	(NT)	(NT)	(NT)	(NT)	+	(NT)	(NT)	(NT)	Family history: OR 3.6 (1.3-10.2)
[4] Feldman, (2001), Canada, 502		X			2/4	13-14 (13.8)	Both	0	0	0	(NT)	(NT)	(NT)	+	(NT)	High growth spurt: OR 3.1 (1.5-6.0)
[16] Franz, (2016), Denmark, 1240	X	X	X		**4/4**	6-12	Both	+		(NT)	(NT)	(NT)	(NT)	(NT)	(NT)	Incidence rate (per 1000): Girls: 0.3 (0.2-0.3), Boys: 0.1 (0.1-0.2) (OR 1.9 (1.4-2.4) (calculated))
[17] Gill, (2014), Australia, 1291	X	X	X		2/4	(14)	Both	+		(NT)	(NT)	(NT)	(NT)	(NT)	(NT)	Females: (X2 = 33.1, p < .001) (OR 2.4 (1.9-3.1) (calculated LBP)) (OR 2.2 (1.6-2.9) (calculated MBP))
[18] Hebert, (2019), Denmark, 1021			X		**4/4**	(9.4)	Both	(NT)	(NT)	(NT)	+	(NT)	(NT)	+	(NT)	Spinal pain (wks), later pubertal status: Tanner stage 2: IRR 1.5 (1.2-2.0) Tanner stage 3: IRR 2.1 (1.5-3.1) Tanner stage 4/5: IRR 3.3 (2.1-5.0) Spinal pain (episodes), pubertal status: Tanner stage 3: 1.4 (1.0-1.8) Tanner stage 4/5: 2.1 (1.4-3.0) Spinal pain (wks): linear growth: IRR 1.2 (1.2-1.2) Spinal pain (episodes), linear growth: IRR 1.1 (1.1-1.2)

Reference	Back pain					Characteristics of study sample		Significant positive (+) or negative (-) associations with back pain								
**Ref, (year of pub), country, pop size**	**MBP**	**LBP**	**Mix**	**?**	**Clear definition of BP (x/4) (Additional file 4)**	**Age range (mean)**	**Sex**	**Female**	**Male**	**Age**	**Pubertal status**	**Family history BP**	**Socioeconomic factors**	**Height**	**History of back pain**	**Significant estimates (95% CI)**
[21] Hestbaek, (2006), Denmark, 9600		X			**4/4**	12-22 (17.3)	Both	(NT)	(NT)	(NT)	(NT)	(NT)	(NT)	(NT)	+	BP in adolescence for BP in adulthood: OR 4.3 (3.5-5.4)
[22] Janssens, (2011), USA/ Denmark 4226				X	1/4	11-15 (11.6) /(13.7)	Both	(NT)	(NT)	(NT)	+	(NT)	(NT)	0	(NT)	Later pubertal status:<br>OR 1.6 (1.3-2.0) (USA)<br>OR 1.3 (1.1-1.6) (Dutch)
[23] Jones, (2009), England, 178		X			2/4	11-14	Both	(NT)	(NT)	(NT)	(NT)	(NT)	(NT)	+	(NT)	Shorter than median height (158cm): RR 2.1 (1.2-3.8)
[24] Jones, (2003), England, 1046		X			2/4	11-14	Both	(NT)	(NT)	(NT)	(NT)	(NT)	(NT)	0	(NT)	
[26] Kroner-Herwig, (2017), Germany, 1522				X	1/4	7-14 (13.4)	Both	+		(NT)	(NT)	(NT)	(NT)	(NT)	(NT)	Female: OR 1.6 (1.2-2.0)
[27] Mattila, (2008), Finland, 57408		X			2/4	14-18 (16.6)	Both		+	(NT)	-	(NT)	(NT)	(NT)	(NT)	Males: HR 3.2 (2.7-3.7)<br>Later pubertal status:<br>HR 0.6 (0.5-0.8) (males)
[28] Mikkonen, (2016), Finland, 1625		X			2/4	15-16	Both	+		(NT)	(NT)	(NT)	(NT)	(NT)	(NT)	Female: Girls 44%, boys 31%<br>(OR 1.7 (1.4-2.1) (calculated))
[29] Mikkonen, (2013), Finland, 1660		X			2/4	15-16	Both	+		(NT)	(NT)	(NT)	(NT)	(NT)	(NT)	Female: prevalence percentage girls 61%, boys 49%<br>(OR 1.6 (1.4-2.0) (calculated)

Reference	Back pain					Characteristics of study sample		Significant positive (+) or negative (-) associations with back pain								
**Ref, (year of pub), country, pop size**	**MBP**	**LBP**	**Mix**	**?**	**Clear definition of BP (x/4) (Additional file 4)**	**Age range (mean)**	**Sex**	**Female**	**Male**	**Age**	**Pubertal status**	**Family history BP**	**Socioeconomic factors**	**Height**	**History of back pain**	**Significant estimates (95% CI)**
[31] Nissinen, (1994), Finland, 859		X			2/4	10-11 (10.8)	Both	0	0	(NT)	(NT)	(NT)	(NT)	+	(NT)	Height: OR 1.2 (1.0-1.5)
[32] Sano, (2015), Japan, 4597		X			2/4	9-10	Both	0	0	+	(NT)	(NT)	(NT)	(NT)	(NT)	Age: 9 yr 10%, 14 yr 25% (OR 2.9 (2.6-3.3) (calculated)
[33] Sjolie, (2004), Norway, 85		X			2/4	14-16 (14.7)	Both	(NT)	(NT)	(NT)	(NT)	(NT)	(NT)	(NT)	+	History of BP: OR 2.7 (1.1-7.1) (ever) OR 9.1 (3.0-27.2) (>7days)
[34] Smith, (2017), Australia, 1088		X			3/4	(14)	Both	+		(NT)	(NT)	(NT)	+	(NT)	+	Females: OR: 2.0 (1.2- 3.0) (no previous BP) OR 7.7 (4.7, 12.6) (previous BP) Higher socioeconomic index: OR 0.8 (0.7-1.0) History of BP: OR 7.7 (4.7-12.6)(girl)
[35] Szita, (2018), Hungary, 952	X	X	X		3/4	7-16 (11.0)	Both	0	0	+	(NT)	+	(NT)	(NT)	(NT)	Older age: OR 1.5 (1.1-2.3) Family history: OR 2.1 (1.4-3.1)
[36] Szpalski, (2002), Belgium, 287		X			1/4	9-12	Both	(NT)	(NT)	(NT)	(NT)	+	(NT)	(NT)	(NT)	Family history: OR 2.0 (1.1-4.0)
Pub: Publication, (NT): Not tested, (NR): Included in study but not reported, + significant positive association, - significant negative association, 0: tested but non-significant estimate, OR: odds ratio, PR: prevalence ratio, RR: relative risk																

Back pain, sample characteristics and associations between back pain and some bidirectional variables. Prospective Studies

Reference	Back Pain					Characteristics of study sample		Significant positive (+) or negative (-) associations with back pain										
Reference (year of pub), country, pop size	MBP	LBP	Mix	?	Clear definition of BP (x/4) (Additional file 4)	Age range (mean)	Sex	BMI	Muscle strength	Flexibility Tightness	Posture	Physical activity/ work	Screen time	Poor Sleep	Carry Bags	Smoke	Illness/ Psychosocial	Significant estimates (95% CI)
[9] Aartun, (2016), Denmark, 625	X	X	X		3/4	11-13	Both	(NT)	(NT)	(NT)	(NT)	0	(NT)	(NT)	(NT)	(NT)	(NT)	
[10] Auvinen, (2010), Finland, 1773		X			2/4	15-16	Both	(NT)	(NT)	(NT)	(NT)	(NT)	(NT)	+	(NT)	(NT)	(NT)	Insufficient Sleep: OR 2.9 (1.7-5.2) (girls) OR 2.4 (1.3-4.5) (boys)
[11] Balague´, (2010), Switzerland, 95		X			2/4	13-14 (14.0)	Male	+	(NT)	(NT)	(NT)	+	(NT)	(NT)	(NT)	(NT)	(NT)	Higher BMI: OR 1.3 (1.0-1.5) Playing sport: OR 9.5 (1.9-48.2)
[12] Deere, (2012), England, 3378	X	X			2/4	17 (17.8)	Both	0	(NT)	(NT)	(NT)	(NT)	(NT)	(NT)	(NT)	(NT)	(NT)	
[13] Feldman, (2002), Canada, 502		X			2/4	13-14 (13.8)	Both	(NT)	(NT)	(NT)	(NT)	+	(NT)	(NT)	(NT)	(NT)	(NT) 0	White collar work: OR 4.9 (1.7-14.2)
[4] Feldman, (2001), Canada, 502		X			2/4	13-14 (13.8)	Both	(NT)	(NT)	+	(NT)	0	(NT)	(NT)	(NT)	+	(NT)	Tight hamstrings: OR 1.1 (1.0-1.1) Smoking: OR 2.2 (1.4-3.5)
[14] Feldman, (1999), Canada, 502		X			2/4	13-14 (13.8)	Both	(NT)	(NT)	(NT)	(NT)	(NT)	(NT)	(NT)	(NT)	+	(NT)	Smoking: OR 2.4 (1.3-6.0)
[15] Franz, (2017), Denmark, 1205	X	X	X		**4/4**	6-12	Both	(NT)	(NT)	(NT)	(NT)	+	(NT)	(NT)	(NT)	(NT)	(NT)	Vigorous intensity physical activity: OR 1.2 (1.0-1.4) (diagnostic spinal pain) OR 1.3 (1.0-1.5) (traumatic)

**Reference**	**Back Pain**					**Characteristics of study sample**		**Significant positive (+) or negative (-) associations with back pain**										
**Reference (year of pub), country, pop size**	**MBP**	**LBP**	**Mix**	**?**	**Clear definition of BP (x/4)** **(Additional file 4)**	**Age range (mean)**	**Sex**	**BMI**	**Muscle strength**	**Flexibility Tightness**	**Posture**	**Physical activity/ work**	**Screen time**	**Poor Sleep**	**Carry Bags**	**Smoke**	**Illness/ Psychosocial**	**Significant estimates (95% CI)**
[16] Franz, (2016), Denmark, 1240	X	X	X		**4/4**	6-12	Both	(NT)	(NT)	(NT)	(NT)	0	(NT)	(NT)	(NT)	(NT)	(NT)	
[17] Gill, (2014), Australia, 1291	X	X	X		2/4	(14)	Both	(NT)	(NT)	(NT)	(NT)	(NT)	(NT)	(NT)	(NT)	+	(NT)<br>0	Smoking: OR 3.1 (1.1-9.2) (MB)<br>OR: 1.8 (1.2-2.8) (BP)
[19] Hestbaek, (2006), Denmark, 9600		X			**4/4**	12-22 (17.3)	Both	0	(NT)	(NT)	(NT)	(NT)	(NT)	(NT)	(NT)	+	(NT)	Smoking: OR 1.7 (1.4-2.1)
[20] Hestbaek, (2006), Denmark, 9600		X			**4/4**	12-22 (17.3)	Both	(NT)	(NT)	(NT)	(NT)	(NT)	(NT)	(NT)	(NT)	(NT)	+	Asthma: OR 1.4 (1.1-1.7) (female)<br>Headache: OR 1.6 (1.1-2.1) (female)<br>OR 2.4 (1.2-4.7) (male)
[23] Jones, (2009), England, 178		X			2/4	11-14	Both	(NT)	(NT)	(NT)	(NT)	(NT)	(NT)	(NT)	(NT)	(NT)	0<br>+	High level of peer problems: RR 2.3 (1.3-4.2)
[24] Jones, (2003), England, 1046		X			2/4	11-14	Both	0	(NT)	(NT)	(NT)	+	0	(NT)	0	(NT)	+<br>+	High level of sports activity (>18hr/wk):<br>RR 1.6 (1.1-2.3)<br>Part-time work:<br>RR 1.5 (1.1-2.1)<br>Abdominal pain: RR 1.8 (1.1-3.0)<br>High level of psychological factors:<br>RR 1.6 (1.1-2.3)
[25] Kanchanomai, (2015), Thailand, 524		X			2/4	18-25 (19.4)	Both	(NT)	(NT)	+	+	(NT)	(NT)	(NT)	(NT)	(NT)	(NT)	Tight quad muscle:<br>OR 1.7 (1.1-2.8)<br>No LB support:<br>OR 1.7 (1.2-2.6)<br>OR 2.9 (1.1-3.5) (persistent LBP)

Reference	Back Pain					Characteristics of study sample		Significant positive (+) or negative (-) associations with back pain										
**Reference (year of pub), country, pop size**	**MBP**	**LBP**	**Mix**	**?**	**Clear definition of BP (x/4)** (Additional file 4)	**Age range (mean)**	**Sex**	**BMI**	**Muscle strength**	**Flexibility Tightness**	**Posture**	**Physical activity/ work**	**Screen time**	**Poor Sleep**	**Carry Bags**	**Smoke**	**Illness/ Psychosocial**	**Significant estimates (95% CI)**
[26] Kroner-Herwig, (2017), Germany, 1522				X	1/4	7-14 (13.4)	Both	(NT)	(NT)	(NT)	(NT)	(NT)	(NT)	(NT)	(NT)	(NT)	+<br>0	Headache: OR 2.4 (1.8-3.1)
[27] Mattila, (2008), Finland, 57408		X			2/4	14-18 (16.6)	Both	(NT)	(NT)	(NT)	(NT)	(NT)	(NT)	(NT)	(NT)	+	(NT)	Daily smoking: HR 1.6 (1.4-1.9)
[28] Mikkonen, (2016), Finland, 1625		X			2/4	15-16	Both	0	(NT)	(NT)	(NT)	(NT)	(NT)	(NT)	(NT)	(NT)	(NT)<br>+	Externalising behaviour: RR: 1.5 (1.3-1.7) (boys) RR: 1.4 (1.3-1.5) (girls) RR: 3.6 (1.5-8.5) (girls 18)
[29] Mikkonen, (2013), Finland, 1660		X			2/4	15-16	Both	+	(NT)	(NT)	(NT)	(NT)	(NT)	(NT)	(NT)	(NT)	(NT)	BMI: RR 1.1 (1.0-1.2) (girls) RR: 1.1 (1.0-1.3) (boys)
[30] Mikkonen, (2008), Finland, 1987		X			2/4	15-16	Both	(NT)	(NT)	(NT)	(NT)	(NT)	(NT)	(NT)	(NT)	+	(NT)	Smoking: OR 2.5 (1.4-4.5) (females)
[31] Nissinen, (1994), Finland, 859		X			2/4	10-11 (10.8)	Both	0	(NT)	(NT)	(NT)	(NT)	(NT)	(NT)	(NT)	(NT)	(NT)	
[32] Sano, (2015), Japan, 4597		X			2/4	9-10	Both	+	(NT)	(NT)	(NT)	(NT)	(NT)	(NT)	(NT)	(NT)	(NT)	High BMI range: OR 2.9 (1.7-5.1) (9 yr) OR 2.2 (1.4-3.5) (10 yr) OR 1.6 (1.2-2.1) (13 yr)
[33] Sjolie, (2004), Norway, 85		X			2/4	14-16 (14.7)	Both	(NT)	(NT)	(NT)	+	+	0	(NT)	(NT)	(NT)	(NT)	Provoked by sitting: OR 3.8 (1.3-11.3) Provoked by manual work: OR 9.2 (2.9-28.8)

Reference	Back Pain					Characteristics of study sample		Significant positive (+) or negative (-) associations with back pain										
**Reference (year of pub), country, pop size**	**MBP**	**LBP**	**Mix**	**?**	**Clear definition of BP (x/4) (Additional file 4)**	**Age range (mean)**	**Sex**	**BMI**	**Muscle strength**	**Flexibility Tightness**	**Posture**	**Physical activity/ work**	**Screen time**	**Poor Sleep**	**Carry Bags**	**Smoke**	**Illness/ Psychos ocial**	**Significant estimates (95% CI)**
[34] Smith, (2017), Australia, 1088		X			3/4	(14)	Both	(NT)	+	(NT)	+	+	(NT)	(NT)	(NT)	(NT)	(NT)<br>+	Poor back muscle endurance:<br>OR 1.9 (1.2-3.0)<br>Non-neutral standing posture:<br>OR 2.2 (1.3-3.6)<br>Increased physical activity:<br>OR 1.9 (1.2-2.8)<br>High levels of aggressive behaviour:<br>OR 1.4 (1.2-1.6)<br>High levels of somatic complaints:<br>OR 1.3 (1.1-1.5)
[35] Szita, (2018), Hungary, 952	X	X	X		3/4	7-16 (11.0)	Both	(NT)	(NT)	(NT)	+	0	+	+	0	(NT)	(NT)	Increased TV time:<br>OR 2 (1.4-2.9)<br>Uncomfortable school desk: OR 6.0 (3.7-9.7)<br>Frequent sleeping problems:<br>2.2 (1.7-3.8)
[36] Szpalski, (2002), Belgium, 287		X			1/4	9-12	Both	(NT)	(NT)	(NT)	(NT)	(NT)	(NT)	+	+	(NT)	(NT)	Quality of sleep:<br>OR 1.2 (1.1-1.4)<br>Heavy school satchel:<br>OR 2.2 (1.0-4.8)
[37] Tobias, (2013), UK, 2901	X	X			3/4	13 (13.8)	Both	(NT)	(NT)	0	(NT)	(NT)	(NT)	(NT)	(NT)	(NT)	(NT)	

Pub: Publication, (NT): Not tested, (NR): Included in study but not reported, + significant positive association, - significant negative association, 0: tested but non-significant estimate, OR: odds ratio, PR: prevalence ratio, RR: relative risk
