# Supplementary material for: Potential risk factors and triggers for back pain in children and young adults. A scoping review, part II: unclear or mixed types of back pain
Source: Chiropr Man Therap. 2019 Nov 19;27:61. doi: 10.1186/s12998-019-0281-8 (PMC6862810; doi:10.1186/s12998-019-0281-8)
Supplement: Supplementary file 3 — Additional file 3. CROSS-SECTIONAL STUDIES reporting factors that are associated with back pain. Table summarising included cross-sectional study. [file 12998_2019_281_MOESM3_ESM.pdf]

Additional file 3: CROSS-SECTIONAL STUDIES reporting factors that are associated with back pain.

Back pain, sample characteristics and associations between back pain and temporal precursor variables. Cross-sectional studies

**Reference**	**Back pain**					**Characteristics of study sample**		**Significant positive (+) or negative (-) associations with back pain**							
**Reference, (year of pub), country, pop size**	**MBP**	**LBP**	**Mix**	**?**	**Clear definition of BP (x/4)** (Additional file 5)	**Age range** (mean)	**Sex**	**Female**	**Male**	**Age**	**Pubertal status**	**Family history BP**	**Socioeconomic factors**	**Height**	**Significant estimates (95% CI)**
[38] Aggarwal, (2013) India, 160		X			3/4	17-25	Both	(NT)	(NT)	(NT)	(NT)	+	(NT)	(NT)	Family history: OR 2.6 (1.4-5.9)
[39] Andersen, (2006), Denmark, 9413			X		2/4	(17)	Both	+		(NT)	(NT)	(NT)	(NT)	(NT)	Female: OR 1.7 (1.5-2.0)
[40] Balague´, (1994), Switzerland, 1755		X			1/4	8-16	Both	(NT)	(NT)	(NT)	(NT)	+	(NT)	(NT)	Family history: OR 2.1
[41] Bejia, (2005), Tunisia, 622		X			**4/4**	11-19	Both	0	0	0	(NT)	+	(NT)	0	Family history: OR 3.8 (2.9-5.9)
[42] Cakmak, (2004), Turkey, 1527		X			3/4	17-26	Both	+		+	(NT)	(NT)	(NT)	(NT)	Female OR: 1.3 (1.4-3.3)<br>Age: 17 yr: OR 1 (index)<br>21 yr OR: 2.2 (1.2-4.2)<br>23 yr OR: 3.2 (1.7-6.2)<br>24 yr OR: 2.8 (1.5-5.3)<br>25+ yr OR: 3.1 (1.4-6.7)
[43] Dianat, (2017), Iran, 1611		X			3/4	11-14	Both	+		0	(NT)	+	(NT)	(NT)	Female: OR 1.5 (1.2–1.8)<br>Family History: OR 1.8 (1.4–2.4)
[44] Dianat, (2014), Iran, 586		X			3/4	12-14	Both	+		0	(NT)	(NT)	(NT)	(NT)	Female: OR 2.2 (1.4-3.3)

**Reference**	**Back pain**					**Characteristics of study sample**		**Significant positive (+) or negative (-) associations with back pain**							
**Reference, (year of pub), country, pop size**	**MBP**	**LBP**	**Mix**	**?**	**Clear definition of BP (x/4)** **(Additional file 5)**	**Age range** **(mean)**	**Sex**	**Female**	**Male**	**Age**	**Pubertal status**	**Family history BP**	**Socioeconomic factors**	**Height**	**Significant estimates (95% CI)**
[45] Diepenmaat, (2006), Netherlands, 3485		X			2/4	12-16	Both	+		(NT)	(NT)	(NT)	(NT)	(NT)	Female: OR 1.5 (1.1-1.9)
[46] Erne, (2011), Switzerland, 189		X			2/4	10-13	Both	0	0	+	(NT)	0	(NT)	(NT)	Younger age: OR 0.2 (0.1-0.6)
[47] Fernandes, (2015), Brazil, 1461		X			3/4	10-14	Both	+		+	(NT)	(NT)	(NT)	(NT)	Female: OR 1.5 (1.1-1.9)<br>Age: 10-11 yr: OR 1 (index)<br>12-14 yr: OR 1.1 (1.1-1.3)
[48] Ganesan, (2017), India, 1355		X			1/4	20-29	Both	0	0	0	(NT)	+	(NT)	(NT)	Family history: Standard error 0.2 (1.4, 2.7)<br>(OR 1.5 (1.1-1.9) calculated)
[49] Ghandour, (2004), USA, 8350				X	2/4	11-19	Both	NR	NR	NR	(NT)	(NT)	0	(NT)	
[50] Gilkey, (2010), USA, 963				X	1/4	18-22	Both	-		0	(NT)	(NT)	0	0	Female: OR 0.6 (0.4-0.8)
[51] Graup, (2014), Brazil, 1455		X			2/4	10-17	Both	+		+	(NT)	(NT)	0	(NT)	Female: OR 2.4 (1.7-3.3)<br>Older age: OR 1.1 (1.1-1.2)
[52] Gunzburg, (1999), Belgium, 392		X			2/4	8-11	Both	0	0	(NT)	(NT)	(NT)	(NT)	(NT)	

Reference	Back pain					Characteristics of study sample		Significant positive (+) or negative (-) associations with back pain							
**Reference, (year of pub), country, pop size**	**MBP**	**LBP**	**Mix**	**?**	**Clear definition of BP (x/4)** (Additional file 5)	**Age range** (mean)	**Sex**	**Female**	**Male**	**Age**	**Pubertal status**	**Family history BP**	**Socioeconomic factors**	**Height**	**Significant estimates (95% CI)**
[53] Haag, (2016), New Zealand, 1110			X		3/4	15-19	Both	+		+	(NT)	(NT)	(NT)	(NT)	Female: OR 1.5 (1.0-2.1)<br>Age 15 yr: OR 1 (index)<br>16/17 yr OR 1.7 (1.2-2.3)<br>18/19 yr: OR 1..8 (1.2-2.8)
[54] Harreby, (1999), Denmark, 1389		X			**4/4**	13-16	Both	+		+	(NT)	(NT)	(NT)	(NT)	Female: OR 2.1 (1.6-2.9)<br>Older age 14 to 15 yr: 6.4%
[55] Hestbaek, (2008), Denmark, 4771		X			3/4	12-18	Both	(NT)	(NT)	(NT)	(NT)	(NT)	+	(NT)	Higher social class: OR 0.9 (0.8-0.9)
[56] Hulsegge, (2011), Netherlands, 2638				X	1/4	10-12	Both	(NT)	(NT)	(NT)	0	(NT)	0	0	
[57] Jones, (2004), England, 1326		X			2/4	12-15	Both	(NT)	(NT)	(NT)	(NT)	0	(NT)	(NT)	
[58] Kaspiris, (2010), Greece, 153		X			3/4	7-14	Both	+		+	(NT)	+	(NT)	+	Female: OR 1.4 (1.0-2.1) (calculated)<br>Older age: OR 1.2<br>Family history: OR 1.7
[59] Kovacs, (2003), Spain, 7048		X			3/4	13-15	Both	+		(NT)	(NT)	(NT)	(NT)	(NT)	Female: OR 1.1 (1.1-1.2)
[60] Kristensen, (2001), Norway, 190		X			3/4	(15)	Both	(NT)	(NT)	(NT)	(NT)	0	(NT)	(NT)	

Reference	Back pain					Characteristics of study sample		Significant positive (+) or negative (-) associations with back pain							
**Reference, (year of pub), country, pop size**	**MBP**	**LBP**	**Mix**	**?**	**Clear definition of BP (x/4)** (Additional file 5)	**Age range** (mean)	**Sex**	**Female**	**Male**	**Age**	**Pubertal status**	**Family history BP**	**Socioeconomic factors**	**Height**	**Significant estimates (95% CI)**
[61] Kristjansdottir, (2002), Iceland, 2173				X	0/4	11-16	Both	0	0	+	(NT)	(NT)	0	(NT)	Older age: r 0.2
[62] Leboeuf-Yde, (2002), Denmark, 806			X		3/4	8-16	Both	(NT)	(NT)	(NT)	(NT)	(NT)	+<br>0	(NT)	Children parental low level of education: OR 1.8 (1.1-2.0) (no significant association in adolescence)
[63] LeResche, (2005), USA, 3101				X	1/4	11-17	Both	0	0	(NT)	+	(NT)	(NT)	(NT)	Later pubertal status:<br>OR 2.0 (girls)<br>OR 1.9 (boys)
[64] Masiero, (2008), Italy, 7542		X			3/4	13-16	Both	+		(NT)	(NT)	+	(NT)	(NT)	Female: OR 1.9 (1.7-2.2)<br>Family history: OR 1.8 (1.5-2.0)
[65] Mattila, (2008), Finland, 7040		X			2/4	18-29	Males	(NT)	(NT)	+	(NT)	(NT)	(NT)	(NT)	Age 17/18 yr: OR: 1 (index)<br>21+ yr: OR 1.6 (1.2-2.1)
[66] Minghelli, (2014), Portugal, 966		X			3/4	10-16	Both	+		+	(NT)	(NT)	(NT)	(NT)	Females: OR 2.1 (1.6-2.7)<br>Age 10-12 yr: OR 1 (index)<br>13-16 yr: 1.5 (1.2-2.0)
[67] Mohseni-Bandpei, (2007), Iran, 4813		X			2/4	11-14	Both	0	0	-	(NT)	(NT)	(NT)	(NT)	Age: OR 0.5 (0.4-0.6)
[68] Ng, (2014), Australia, 265		X			2/4	14-16	Both		+	(NT)	(NT)	(NT)	(NT)	(NT)	Prevalence percentage<br>Male: Lifetime 94%, point 65%<br>Female: Lifetime 78%, point 53%<br>(Females: Lifetime: OR 0.2 (0.1-0.5), point: OR 0.3 (0.2-0.5) calculated)

Reference	Back pain					Characteristics of study sample		Significant positive (+) or negative (-) associations with back pain							
**Reference, (year of pub), country, pop size**	**MBP**	**LBP**	**Mix**	**?**	**Clear definition of BP (x/4)** (Additional file 5)	**Age range** (mean)	**Sex**	**Female**	**Male**	**Age**	**Pubertal status**	**Family history BP**	**Socioeconomic factors**	**Height**	**Significant estimates (95% CI)**
[69] Noll, (2016), Brazil, 1597				X	2/4	11-16	Both	+		0	(NT)	+	0	(NT)	Female: PR 1.1 (1.1-1.2)<br>Family history: PR 1.2 (1.2-1.3)
[70] Noll, (2016), Brazil, 361				X	2/4	14-20	Both	+		0	(NT)	+	+	(NT)	Female: PR 1.2 (1.1-1.3)<br>Family history: PR 1.2 (1.1-1.3)<br>Ethnicity: White: PR: 1<br>Asian: PR 1.2 (1.1-1.4)<br>Indigenous: PR 1.4 (1.3-1.5)
[71] Onofrio, (2012), Brazil, 1233		X			3/4	13-19	Both	0	0	0	(NT)	(NT)	+	(NT)	Non-white: PR 1.4 (1.0-1.9)
[72] Pasanen, (2016), Finland, 401		X			3/4	13-17	Both	0	0	+	(NT)	+	(NT)	(NT)	Age (per year): OR 1.2 (1.1-1.4)<br>Family history: OR 2.0 (1.2-3.3)
[73] Prista, (2004), Mozambique, 204		X			3/4	11-16	Both	0	0	0	(NT)	(NT)	+	(NT)	Living in peripheral centre: OR 1<br>Living in wealthier urban centre OR: 3.1
[74] Rodrigues-Oviedo, (2012), Spain, 1403				X	1/4	12-17	Both	+		+	(NT)	(NT)	(NT)	(NT)	Female: OR 1.6 (1.3-2.1)<br>Older age: OR 1.2 (1.1-1.3)
[75] Scarabottolo, (2017), Brazil, 1011		X			3/4	10-17	Both	0	0	+	(NT)	(NT)	0	(NT)	Older age: older 25.1%, younger adolescents 12.4%
[76] Shehab, (2004), Kuwait, 400		X			2/4	10-18	Both	+		(NT)	(NT)	(NT)	(NT)	(NT)	Female: prevalence percentage 64.7%<br>males: prevalence percentage 50.8%<br>(Females: OR: 1.8 (1.2-2.7) calculated)

Reference	Back pain					Characteristics of study sample		Significant positive (+) or negative (-) associations with back pain							
Reference, (year of pub), country, pop size	MBP	LBP	Mix	?	Clear definition of BP (x/4) (Additional file 5)	Age range (mean)	Sex	Female	Male	Age	Pubertal status	Family history BP	Socioeconomic factors	Height	Significant estimates (95% CI)
[77] Sheir-Neiss (2003), USA, 1126				X	2/4	12-18	Both	+		(NT)	(NT)	(NT)	(NT)	(NT)	Female: OR 2.2 (1.6-2.9)
[78] Shipp, (2007), USA, 2536				X	3/4	14-18	Both	+		(NT)	(NT)	(NT)	(NT)	(NT)	Female: OR 4.6 (1.8-11.7)
[79] Silva, (2016), Brazil, 961			X		3/4	14-19	Both	+		0	(NT)	(NT)	(NT)	(NT)	Female: OR 2.4 (1.9-3.2)
[80] Silva, (2014), Brazil, 343		X			3/4	12-15	Both	0	0	+	(NT)	(NT)	0	(NT)	Age 12 yr: OR 1 (index)<br>14 yr: OR 1.3 (1.1-1.7)
[81] Skaggs, (2006), USA, 1540				x	1/4	10-15	Both	+		-	(NT)	(NT)	(NT)	(NT)	Female: 43%, Male: 32%<br>(Female: OR: 1.6 (1.3-2.0) calculated)<br>Younger age: OR 1.5(male), OR 1.4(female)
[82] Turk, (2011), Slovenia, 190		X			2/4	11-15	Both	(NT)	(NT)	0	(NT)	(NT)	(NT)	0	
[83] Van Gent, (2003), Netherlands, 745				X		12-14	Both	+		0	(NT)	(NT)	+	(NT)	Female: 54%, Males 39%<br>(females: OR: 1.8 (1.3-2.4) calculated)<br>Residence: 52% (city), 43% (village)
[84] Viry, (1999), France 123				X	2/4	13-15	Both	+		(NT)	(NT)	(NT)	(NT)	(NT)	Female: OR 2.7 (1.2-6.1)

Reference	Back pain					Characteristics of study sample		Significant positive (+) or negative (-) associations with back pain							
**Reference, (year of pub), country, pop size**	**MBP**	**LBP**	**Mix**	**?**	**Clear definition of BP (x/4)** (Additional file 5)	**Age range** (mean)	**Sex**	**Female**	**Male**	**Age**	**Pubertal status**	**Family history BP**	**Socioeconomic factors**	**Height**	**Significant estimates (95% CI)**
[85] Watson, (2003), Great Britain, 1446		X			3/4	11-14	Both	+		+	(NT)	(NT)	(NT)	(NT)	Female: 28%, Males 19%<br>Girls 11 yr: 18%, 14 yr: 34%<br>Boys 11 yr: 14%, 14 yr: 25%
[86] Wedderkopp, (2005), Denmark, 254	X	X	X		3/4	8-16	Both	(NT)	(NT)	(NT)	+	(NT)	(NT)	(NT)	Later pubertal status:<br>Stage 1: OR 1 (index)<br>Stage 4: OR 2.0 (1.3-3.5)<br>Stage 5: OR 2.1 (1.1-4.1)
[87] Wedderkopp, (2001), Denmark, 806	X	X	X		3/4	8-16	Both	0	0	(NT)	(NT)	(NT)	(NT)	(NT)	
[88] Wirth, (2015), Switzerland, 412	X	X	X		3/4	10-16	Both	0	0	+	(NT)	0	(NT)	(NT)	Older age: OR 1.3 (1.1-1.7)
[89] Wirth, (2013), Switzerland, 434	X	X	X		3/4	6-16	Both	+		+	(NT)	+	(NT)	(NT)	Female: OR 1.9 (1.3-3.0)<br>Older age: OR 1.3 (1.2-1.4)<br>Family history: 2.3 (1.2-4.7)
[90] Yao, (2012), China, 1214		X			3/4	13-17	Both	(NT)	(NT)	0	(NT)	+	(NT)	0	Family history: 2.6 (1.9-3.6)

(NT): Not tested, (NR): Included in study but not reported, *+ significant positive association, *- significant negative association, 0: tested but non-significant estimate, OR: odds ratio, SE: standard error, PR: prevalence ratio, RR: relative risk
