# Supplementary material for: Potential risk factors and triggers for back pain in children and young adults. A scoping review, part II: unclear or mixed types of back pain
Source: Chiropr Man Therap. 2019 Nov 19;27:61. doi: 10.1186/s12998-019-0281-8 (PMC6862810; doi:10.1186/s12998-019-0281-8)
Supplement: Supplementary file 4 — Additional file 4. Clarity of definitions of Back pain: Prospective studies. Table summarising the clarity of the definitions of back pain in included prospective studies. [file 12998_2019_281_MOESM4_ESM.pdf]

Additional file 4: Clarity of definitions of Back pain: Prospective studies

	**Area of BP (1 point)**	**Recall period (1 point)**						**Type (1 point)**	**Severity described**	**Consequences reported**	**Attempted to collect valid data (1 point)**	**Conclusion**
**Ref (year of pub)**	**Location**	**Now**	**Past week**	**Past month**	**Past year**	**> 1 year**	**pain ever**	**-1st ever**<br>**-Episodic**<br>**-Ongoing**<br>**-?**		**-Seek care**<br>**-Downtime**<br>**-Disability**		**Clear definition of BP (x/4)**
[9] Aatun, (2016)	MB/LB						X	-<br>-<br>-<br>-?	No	-<br>-<br>-	Diagram used, pilot study of the questionnaire	3/4
[10] Auvinen, (2010)	LB			X (6 mth)				-<br>-<br>-<br>-?	No	-Seek care<br>-<br>-	Diagram used	2/4
[11] Balague´, (2010)	LB		X	X	X			-<br>-<br>-<br>-?	No	-Seek care<br>-<br>-Disability	Used diagram	2/4
[12] Deere, (2012)	MB/LB	X		X (6 mth)				-<br>-<br>-<br>-?	Yes	-<br>-<br>-	Used a pre-validated questionnaire. Diagram used.	3/4
[13] Feldman, (2002)	LB			X (6 mth)				-<br>-<br>-<br>-?	No	-<br>-<br>-	NR	2/4
[4] Feldman, (2001)	LB			X (6 mth)				-<br>-<br>-<br>-?	No	-Seek care<br>-<br>-Disability	NR	2/4
[14] Feldman, (1999)	LB			X (6 mth)				-<br>-<br>-<br>-?	No	-<br>-<br>-	NR	2/4
[15] Franz, (2017)	MB/LB		X					-<br>-Episodic<br>-Ongoing<br>-	No	-<br>-<br>-	Weekly SMS tracking	**4/4**

	**Area of BP (1 point)**	**Recall period (1 point)**						**Type (1 point)**	**Severity described**	**Consequences reported**	**Attempted to collect valid data (1 point)**	**Conclusion**
**Ref (year of pub)**	**Location**	**Now**	**Past week**	**Past month**	**Past year**	**> 1 year**	**pain ever**	**-1st ever<br>-Episodic<br>-Ongoing<br>-?**		**-Seek care<br>-Downtime<br>-Disability**		**Clear definition of BP (x/4)**
[16] Franz, (2016)	MB/LB		X					-<br>-Episodic<br>-Ongoing<br>-	No	-<br>-<br>-	Weekly SMS tracking	**4/4**
[17] Gill, (2014)	MB/LB			X				-<br>-<br>-<br>-?	No	-<br>-<br>-	NR	2/4
[18] Hebert, (2019)	MB/LB		X					-<br>-Episodic<br>-Ongoing<br>-	No	-<br>-<br>-	SMS tracking	**4/4**
[19] Hestbaek, (2006)	LBP				X			-<br>-Episodic<br>-Ongoing<br>-	No	-<br>-<br>-	Used pre-validated questionnaire. Diagram used.	**4/4**
[20] Hestbaek, (2006)	LBP				X			-<br>-Episodic<br>-Ongoing<br>-	No	-<br>-<br>-	Used pre-validated questionnaire. Diagram used.	**4/4**
[21] Hestbaek, (2006)	LBP				X			-<br>-Episodic<br>-Ongoing<br>-	No	-<br>-<br>-	Used pre-validated questionnaire. Diagram used.	**4/4**
[22] Janssens, (2011)	?			X (3 mth)				-<br>-<br>-<br>-?	No	-<br>-<br>-	NR	1/4
[23] Jones, (2009)	LB			X				-<br>-<br>-<br>-?	No	-<br>-<br>-	Diagram used	2/4
[24] Jones, (2003)	LB			X				-<br>-<br>-<br>-?	No	-<br>-<br>-	Diagram used	2/4

	Area of BP<br>(1 point)	Recall period<br>(1 point)						Type<br>(1 point)	Severity described	Consequences reported	Attempted to collect valid data<br>(1 point)	Conclusion
**Ref<br>(year of pub)**	**Location**	**Now**	**Past week**	**Past month**	**Past year**	**> 1 year**	**pain ever**	**-1st ever<br>-Episodic<br>-Ongoing<br>-?**		**-Seek care<br>-Downtime<br>-Disability**		**Clear definition of BP<br>(x/4)**
[25] Kanchanomai, (2015)	LB			X (3mth)				-<br>-<br>-<br>-?	No	-<br>-<br>-	Diagram used	2/4
[26] Kroner-Herwig (2017)	?			X (6 mth)				-<br>-<br>-<br>-?	No	-<br>-<br>-	NR	1/4
[27] Mattila, (2008)	LB				X			-<br>-<br>-<br>-?	No	-Seek care<br>-<br>-	Used ICD-10 diagnosis codes	2/4
[28] Mikkonen, (2016)	LB			X (6 mth)				-<br>-<br>-<br>-?	No	-Seek care<br>-<br>-	NR	2/4
[29] Mikkonen, (2013)	LB			X (6 mth)				-<br>-<br>-<br>-?	No	-Seek care<br>-<br>-	Diagram used	2/4
[30] Mikkonen, (2008)	LB			X (6 mth)				-<br>-<br>-<br>-?	No	-Seek care<br>-<br>-	Diagram used	2/4
[31] Nissinen, (1994)	LB	X	X	X	X		X	-<br>-<br>-<br>-?	No	-Seek care<br>-<br>-	Diagram used	2/4
[32] Sano, (2015)	LB	X					X	-<br>-<br>-<br>-?	No	-<br>-<br>-	Diagram used	2/4
[33] Sjolie, (2004)	LB				X		X	-<br>-<br>-<br>-?	No	-Seek care<br>-<br>-Disability	Diagram used	2/4

	**Area of BP (1 point)**	**Recall period (1 point)**						**Type (1 point)**	**Severity described**	**Consequences reported**	**Attempted to collect valid data (1 point)**	**Conclusion**
**Ref (year of pub)**	**Location**	**Now**	**Past week**	**Past month**	**Past year**	**> 1 year**	**pain ever**	**-1st ever<br>-Episodic<br>-Ongoing<br>-?**		**-Seek care<br>-Downtime<br>-Disability**		**Clear definition of BP (x/4)**
[34] Smith, (2017)	LB			X				-<br>-<br>-<br>-?	No	-Seek care<br>-Downtime<br>-Disability	Used pre-validated questionnaire	3/4
[35] Szita, (2018)	LB/MB			X				-<br>-<br>-<br>-?	No	-Seek care<br>-Downtime<br>-	Pilot study of questionnaire.	3/4
[36] Szpalski, (2002)	LB	?	?	?	?	?	?	-<br>-<br>-<br>-?	Yes	-<br>-<br>-	NR	1/4
[37] Tobias, (2013)	MB/LB			X				-<br>-<br>-<br>-?	Yes	-<br>-<br>-Disability	Used pre-validated questionnaire	3/4

BP: back pain, LB: low back, MB: mid back, NR: not reported, mth: months
