# Supplementary material for: Potential risk factors and triggers for back pain in children and young adults. A scoping review, part II: unclear or mixed types of back pain
Source: Chiropr Man Therap. 2019 Nov 19;27:61. doi: 10.1186/s12998-019-0281-8 (PMC6862810; doi:10.1186/s12998-019-0281-8)
Supplement: Supplementary file 5 — Additional file 5. Clarity of definitions of Back pain: Cross-sectional studies. Table summarising the clarity of the definitions of back pain in included cross-sectional studies. [file 12998_2019_281_MOESM5_ESM.pdf]

Additional file 5: Clarity of definitions of Back pain: Cross-sectional studies

	Area of BP (1 point)	Recall period (1 point)						Type (1 point)	Severity described	Consequences reported	Attempted to collect valid data (1 point)	Conclusion
**Ref (year of pub)**	**Location**	**Now**	**Past week**	**Past month**	**Past year**	**> 1 year**	**pain ever**	**-1st ever<br>-Episodic<br>-Ongoing<br>-?**		**-Seek care<br>-Downtime<br>-Disability**		**Clear definition of BP (x/4)**
[38] Aggarwal, (2013)	LB		X	X	X			-<br>-<br>-<br>-?	Yes	-<br>- Downtime<br>- Disability	Questionnaire based on previous guidelines, pretested in pilot study.	3/4
[39] Andersen, (2006)	LB,<br>MB,	X		X			X	-<br>-<br>-<br>-?	No	- Seek care<br>- Downtime<br>-	NR	2/4
[40] Balague´, (1994)	LB	?	?	?	?	?	?	-<br>-<br>-<br>-?	No	-<br>-<br>-	NR	1/4
[41] Bejia, (2005)	LB		X	X	X			-<br>-Episodic<br>-Ongoing<br>-	No	-Seek care<br>-Downtime<br>-Disability	Used pre-validated questionnaire (kappa: 0.7-1.0)<br>Used diagrams	**4/4**
[42] Cakmak, (2004)	LB			X			X	-<br>-<br>-<br>-?	Yes	-<br>-<br>- Disability	Pilot study of questionnaire: ICC +1 (perfect agreement)	3/4
[43] Dianat, (2017)	LB			X				-<br>-<br>-<br>-?	Yes	- Seek care<br>- Downtime<br>- Disability	Pilot study on questionnaire prior. Test-retest stability kappa: 0.72-0.96)<br>Used diagrams	3/4
[44] Dianat, (2014)	LB			X				-<br>-<br>-<br>-?	No	-<br>-<br>-	Modified pre-validated questionnaire. Pilot study on questionnaire. Test-retest: phi coefficients: 0.72-0.91)<br>Used diagrams	3/4
[45] Diepenmaat, (2006)	LB			X				-<br>-<br>-<br>-?	No	-<br>-<br>-	Used diagram	2/4

	Area of BP (1 point)	Recall period (1 point)						Type (1 point)	Severity described	Consequences reported	Attempted to collect valid data (1 point)	Conclusion
Ref (year of pub)	Location	Now	Past week	Past month	Past year	> 1 year	pain ever	-1st ever<br>-Episodic<br>-Ongoing<br>-?		-Seek care<br>-Downtime<br>-Disability		Clear definition of BP (x/4)
[46] Erne, (2011)	LB			X				-<br>-<br>-<br>-?	No	-<br>-<br>-	Used diagram	2/4
[47] Fernandes, (2015)	LB				X			-<br>-<br>-<br>- ?	No	-<br>-<br>-	Adapted a pre-validated questionnaire<br>Used diagram	3/4
[48] Ganesan, (2017)	LB	?	?	?	?	?	?	-<br>-<br>-<br>-?	Yes	-<br>-<br>- Disability	NR	1/4
[49] Ghandour, (2004)	?		X	X				-<br>-Episodic<br>-Ongoing<br>-	No	-<br>-<br>-	NR	2/4
[50] Gilkey, (2010)	?				X			-<br>-<br>-<br>- ?	No	- Seek care<br>-<br>-	NR	1/4
[51] Graup, (2014)	LB						X	-<br>-<br>-<br>- ?	No	-<br>-<br>-	Diagrams used	2/4
[52] Gunzburg, (1999),	LB						X	-<br>-<br>-<br>- ?	Yes	- Seek care<br>-Downtime<br>-	NR	2/4
[53] Haag, (2016)	Mix				X			-<br>-<br>-<br>- ?	Yes	-<br>-<br>-	Stated used a validated questionnaire, diagram used	3/4
[54] Harreby, (1999)	LB	X	X	X	X		X	-<br>-Episodic<br>-Ongoing<br>-	Yes	-Seek care<br>-Downtime<br>-Disability	Pilot study on questionnaire.<br>Diagram used	4/4

	Area of BP<br>(1 point)	Recall period<br>(1 point)						Type<br>(1 point)	Severity described	Consequences reported	Attempted to collect valid data<br>(1 point)	Conclusion
**Ref<br>(year of pub)**	**Location**	**Now**	**Past week**	**Past month**	**Past year**	**> 1 year**	**pain ever**	**-1st ever<br>-Episodic<br>-Ongoing<br>-?**		**-Seek care<br>-Downtime<br>-Disability**		**Clear definition of BP<br>(x/4)**
[55] Hestbaek, (2008)	LB				X			-<br>-<br>-<br>-?	No	-<br>-<br>-	Used a pre-validated questionnaire	3/4
[56] Hulsegge, (2011)	?				X			-<br>-<br>-<br>- ?	No	-Seek care<br>-<br>-	NR	1/4
[57] Jones, (2004)	LB			X				-<br>-<br>-<br>- ?	No	-<br>-<br>-	Diagrams used	2/4
[58] Kaspiris, (2010)	LB				X			-<br>-<br>-<br>-?	Yes	-<br>-<br>-Disability	Used a pre-validated questionnaire, diagrams used	3/4
[59] Kovacs, (2003)	LB		X				X	-<br>-<br>-<br>-?	No	-Seek care<br>-Downtime<br>-Disability	Use a pre-validated questionnaire	3/4
[60] Kristensen, (2001)	LB				X		X	-<br>-<br>-<br>-?	No	-Seek care<br>-Downtime<br>-Disability	Use pre-validated questionnaire, piloted the questionnaire, used diagram	3/4
[61] Kristjansdottir, (2002)	?	?	?	?	?	?	?	-<br>-<br>-<br>-?	No	-<br>-<br>-	NR	0/4
[62] Leboeuf-Yde, (2002),	Mix	X	X	X				-<br>-<br>-<br>-?	No	-Seek care<br>-Downtime<br>-Disability	NR	3/4
[63] LeResche, (2005)	?			X (3 mth)				-<br>-<br>-<br>-?	Yes	-<br>-<br>-	NR	1/4

	Area of BP<br>(1 point)	Recall period<br>(1 point)						Type<br>(1 point)	Severity described	Consequences reported	Attempted to collect valid data<br>(1 point)	Conclusion
**Ref<br>(year of pub)**	**Location**	**Now**	**Past week**	**Past month**	**Past year**	**> 1 year**	**pain ever**	**-1st ever<br>-Episodic<br>-Ongoing<br>-?**		**-Seek care<br>-Downtime<br>-Disability**		**Clear definition of BP<br>(x/4)**
[64] Masiero, (2008)	LB				X			-<br>-<br>-<br>-?	Yes	-Seek care<br>-<br>-	Assessed for comprehensibility in a pilot study	3/4
[65] Mattila, (2008)	LB						X	-<br>-<br>-<br>-?	No	-Seek care<br>-<br>-	NR	2/4
[66] Minghelli, (2014)	LB	X			X		X	-<br>-<br>-<br>-?	No	-<br>-<br>-	Used a pre-validated questionnaire	3/4
[67] Mohseni-Bandpei, (2007),	LB	X		X	X			-<br>-<br>-<br>-?	No	-<br>-<br>-	NR	2/4
[68] Ng, (2014)	LB	X	X				X	-<br>-<br>-<br>-?	Yes	-<br>-<br>-	Diagram used	2/4
[69] Noll, (2016)	?			X (3 mth)				-<br>-<br>-<br>-?	No	-<br>-<br>-	Used a pre-validated questionnaire	2/4
[70] Noll, (2016)	?			X (3 mth)				-<br>-<br>-<br>-?	No	-<br>-<br>-	Used a pre-validated questionnaire	2/4
[71] Onofrio, (2012)	LB			X				-<br>-<br>-<br>-?	No	-<br>-<br>-	Pilot study of questionnaire, diagram used	3/4
[72] Pasanen, (2016)	LB		X		X			-<br>-<br>-<br>-?	No	-Seek care<br>-Downtime<br>-Disability	Used pre-validated questionnaire	3/4

	Area of BP<br>(1 point)	Recall period<br>(1 point)						Type<br>(1 point)	Severity described	Consequences reported	Attempted to collect valid data<br>(1 point)	Conclusion
**Ref<br>(year of pub)**	**Location**	**Now**	**Past week**	**Past month**	**Past year**	**> 1 year**	**pain ever**	**-1st ever<br>-Episodic<br>-Ongoing<br>-?**		**-Seek care<br>-Downtime<br>-Disability**		**Clear definition of BP<br>(x/4)**
[73] Prista, (2004)	LB			X	X		X	-<br>-Episodic<br>-<br>-	No	-Seek care<br>-<br>-	NR	3/4
[74] Rodrigues-Oviedo, (2012)	?				X			-<br>-<br>-<br>-?	No	-<br>-<br>-	NR	1/4
[75] Scarabottolo, (2017)	LB		X					-<br>-<br>-<br>-?	No	-<br>-<br>-	Used a pre-validated questionnaire, (kappa: 0.57-1.00)	3/4
[76] Shehab, (2004)	LB	?	?	?	?	?	?	-<br>-<br>-?	Yes	-<br>-<br>-Disability	Questionnaire was pre-tested. Diagrams used.	2/4
[77] Sheir-Neiss (2003)	?			X				-<br>-<br>-<br>-?	Yes	-Seek care<br>-Downtime<br>-Disability	Adapted a pre-validated questionnaire. Diagrams used.	2/4
[78] Shipp, (2007)	?			X (9 mth)				-<br>-<br>-?	No	-Seek care<br>-Downtime<br>-Disability	Pre-validated questionnaire used. Diagram used.	3/4
[79] Silva, (2016)	MB/LB			X (6 mth)				-<br>-<br>-<br>-?	No	-Seek care<br>-<br>-	Diagrams used. Use a pre-validated questionnaire.	3/4
[80] Silva, (2014)	LBP				X			-<br>-<br>-<br>-?	No	-<br>-<br>-	Use a pre-validated questionnaire. Diagrams used.	3/4
[81] Skaggs, (2006)	?	?	?	?	?	?	?	-<br>-<br>-<br>-?	Yes	-Seek care<br>-<br>-Disability	Use a pre-validated questionnaire	1/4

	Area of BP (1 point)	Recall period (1 point)						Type (1 point)	Severity described	Consequences reported	Attempted to collect valid data (1 point)	Conclusion
**Ref (year of pub)**	**Location**	**Now**	**Past week**	**Past month**	**Past year**	**> 1 year**	**pain ever**	**-1st ever<br>-Episodic<br>-Ongoing<br>-?**		**-Seek care<br>-Downtime<br>-Disability**		**Clear definition of BP (x/4)**
[82] Turk, (2011)	LB			X (3 mth)				-<br>-<br>-<br>-?	Yes	-Seek care<br>-<br>-Disability	NR	2/4
[83] Van Gent, (2003)	?	?	?	?	?	?	?	-<br>-<br>-<br>-?	No	-Seek care<br>-<br>-Disability	NR	0/4
[84] Viry, (1999)	?	X					X	-<br>-Episodic<br>-Ongoing<br>-	No	-Seek care<br>-Downtime<br>-	NR	2/4
[85] Watson, (2003)	LB			X				-<br>-<br>-<br>-?	No	-<br>-<br>-	Piloted questionnaire. Used diagram	3/4
[86] Wedderkopp, (2005)	MB/LB	X	X	X				-<br>-<br>-<br>-?	No	-Seek care<br>-Downtime<br>-	Pilot study used for questionnaire, diagrams used.	3/4
[87] Wedderkopp, (2001)	MB/LB	x	x	x				-<br>-<br>-<br>-?	No	-Seek care<br>-Downtime<br>-	Pilot study used for questionnaire, diagrams used.	3/4
[88] Wirth, (2015)	MB/LB			X			X	-<br>-Episodic<br>-Ongoing<br>-	yes	-Seek care<br>-Downtime<br>-Disability	Diagram used	3/4
[89] Wirth, (2013)	MB/LB			X			X	-<br>-Episodic<br>-Ongoing<br>-	Yes	-Seek care<br>-Downtime<br>-Disability	NR	3/4

	Area of BP (1 point)	Recall period (1 point)						Type (1 point)	Severity described	Consequences reported	Attempted to collect valid data (1 point)	Conclusion
Ref (year of pub)	Location	Now	Past week	Past month	Past year	> 1 year	pain ever	-1st ever<br>-Episodic<br>-Ongoing<br>-?		-Seek care<br>-Downtime<br>-Disability		Clear definition of BP (x/4)
[90] Yao, (2012)	LB			X (3 mth)				-<br>-<br>-<br>-?	No	-<br>-<br>-	Pilot study used for questionnaire, test=retest coefficient 0.5-0.8)	3/4
BP: back pain, LB: low back, MB: mid back, NR: not reported,												
